# Boosting Hydrogen Photogeneration via Controlled CdS Nucleation on PEI-Modified Graphene Surfaces

**DOI:** 10.3390/molecules31111920

**Published:** 2026-06-02

**Authors:** José J. Chica-Armenteros, Joan Vernet-García, Rubén Cruz-Sánchez, Celeste García-Gallarín, Antonio Peñas-Sanjuán, Manuel Melguizo

**Affiliations:** Departamento de Química Inorgánica y Orgánica, Facultad de Ciencias Experimentales, Universidad de Jaén, 23071 Jaén, Spain; jjchica@ujaen.es (J.J.C.-A.); jvernet@ujaen.es (J.V.-G.); rcruz@ujaen.es (R.C.-S.); cgarcia@ujaen.es (C.G.-G.)

**Keywords:** CdS, graphene, polyethyleneimine, hydrogen production, graphene modification

## Abstract

The performance of CdS-based photocatalysts can be enhanced by incorporating graphene co-catalysts in close contact with the photoactive phase. However, assembling these distinct components remains a bottleneck, as their differing chemical natures often limit effective interfacial interaction when they are synthesized separately. In this work, we present an adaptable PEI-mediated interfacial assembly strategy for promoting the nucleation and growth of nanocrystalline CdS phases on different graphene-based supports within a common, yet support-adapted, approach. Specifically, by functionalizing the surface of various graphene materials with hyperbranched polyethyleneimine (PEI) as a multifunctional interlayer mediator, we achieve controlled CdS formation. This strategy provides a common chemical framework for producing CdS nanocrystals closely associated with the carbon surface, regardless of the substrate. Diverse materials, including low-defect graphene sheets (G-Sheets), graphene nanoplatelets (GNPs), and graphene oxide (GO), were integrated using tailored architectures: noncovalent PDI-anchoring for GNP and G-Sheets and direct covalent functionalization for GO. In the latter case, PEI acts simultaneously as a mild reducing agent, yielding a covalently grafted reduced graphene oxide hybrid (rGO-PEI). XRD patterns confirm comparable CdS crystallinity across all hybrids, while photocatalytic hydrogen evolution measurements reveal a strong dependence on the nature of the graphene support. rGO-PEI@CdS exhibits the highest hydrogen evolution rate (0.44 mmol g^−1^ h^−1^) without any noble-metal cocatalyst, highlighting the role of surface defects and oxygen functionalities in interfacial charge transfer. Thermal treatment of rGO-PEI@CdS enhances activity (average 1.20 mmol g^−1^ h^−1^) but leads to partial deactivation over time. Overall, this study provides an adaptable PEI-mediated framework for integrating diverse graphene-type materials as co-catalysts within CdS-based photocatalytic materials and investigates structure–function relationships in graphene@CdS systems.

## 1. Introduction

Cadmium sulfide (CdS) is a well-established visible-light-responsive semiconductor, widely investigated for photocatalytic hydrogen evolution due to its narrow direct band gap (~2.4 eV) and a conduction band edge that is thermodynamically well-positioned for proton reduction [1,2]. Despite these favorable electronic characteristics, the performance of CdS-based photocatalysts is rarely governed by intrinsic energetics alone. In practice, their solar-to-hydrogen performance and operational stability are severely constrained by sluggish charge-transfer kinetics, rapid electron-hole recombination, and the accumulation of photogenerated carriers at the surface. These processes not only suppress photocatalytic hydrogen production efficiencies, but also accelerate photocorrosion, severely compromising long-term operational stability under continuous irradiation. [3,4].

To overcome these limitations, CdS is commonly integrated with conductive carbon materials capable of promoting charge separation and assisting electron transport away from the semiconductor surface. Among these, graphene-based materials, in particular, are utilized as cocatalyst supports due to their exceptional electrical conductivity and extended π-conjugated networks, which promote electron delocalization and suppress recombination [5,6,7]. However, the beneficial role of graphene-based components in CdS photocatalysis is critically dependent on the establishment of an effective semiconductor/carbon interface. Achieving such contact is challenging via the mixing of individually pre-formed phases due to their differing chemical natures. Furthermore, the term “graphene” encompasses a broad family of carbon materials rather than a single, chemically uniform entity. Low-defect graphene sheets (G-Sheets), graphene nanoplatelets (GNPs), and graphene oxide (GO) exhibit marked differences in surface chemistry, defect density, and electronic structure. These intrinsic differences strongly influence their interfacial coupling with CdS and consequently may affect the efficiency of photoinduced charge separation and transfer processes [8,9].

In this work, we address this challenge by establishing an interfacial assembly framework that enables a comparison of different graphene supports within a common PEI-mediated chemical approach adaptable to the variable nature of the graphene-type support. Central to this strategy is the use of hyperbranched polyethyleneimine (PEI) as a multifunctional chemical tool for CdS formation. Owing to its exceptionally high density of primary, secondary, and tertiary amine groups, PEI exhibits a pronounced polydentate chelating character [10,11,12], enabling strong and multivalent complexation of Cd^2+^ ions and providing local coordination environments that promote CdS nucleation on the surfaces where PEI can be linked. In polymer-mediated syntheses of CdS, such coordination and capping effects are well established to suppress uncontrolled nanocrystal growth and aggregation, yielding highly dispersed semiconductor domains with maximized active surface area [13,14,15]. Here, this behavior is leveraged to promote the formation of nanocrystalline CdS domains on different PEI-modified carbon supports under a comparable interfacial assembly concept.

**Figure 1:**
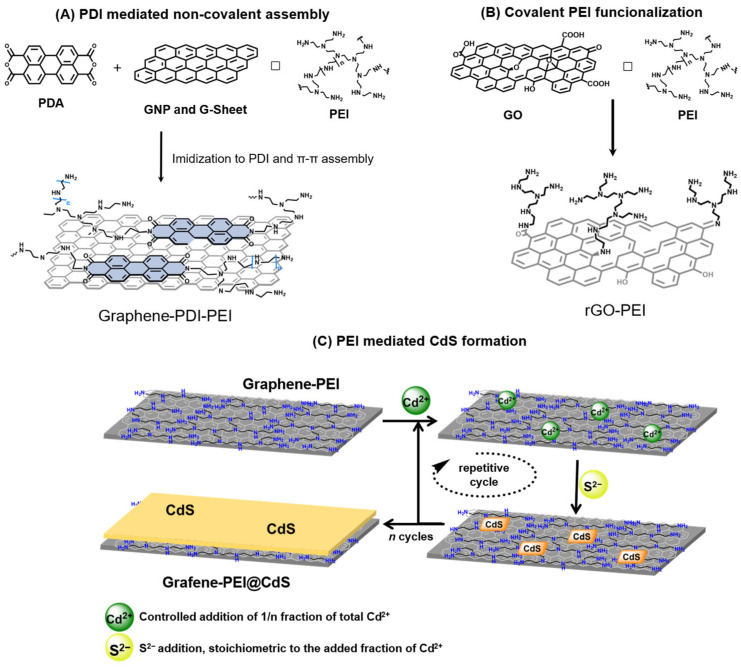
Schematic illustration of the interfacial assembly strategies employed for graphene-based supports. (**A**) PDI-mediated non-covalent functionalization of graphene sheets (G-Sheets) and nanoplatelets (GNPs). (**B**) Covalent PEI functionalization of graphene oxide (GO). (**C**) PEI-mediated CdS formation.

To bridge the polymer-stabilized semiconductor with the carbon lattice, we employ a modular supramolecular approach. For G-Sheets and GNPs, the low abundance of oxygenated functional groups precludes direct covalent grafting of PEI. Therefore, perylenediimide (PDI) aromatic units covalent-bonded to PEI are employed as aromatic anchors that associate with graphene via noncovalent π-π stacking. For the oxygenated surface of GO, a covalent functionalization pathway is employed, allowing direct integration of PEI without the need for aromatic linkers (Figure 1).

By comparing G-Sheet-PDI-PEI@CdS, GNP-PDI-PEI@CdS, and rGO-PEI@CdS hybrids, this study assesses how the identity of the graphene derivative and the nature of the interface influence the photocatalytic hydrogen production. Beyond the specific CdS system, this work establishes the PDI-PEI-carbon architecture as a versatile and transferable platform for photocatalyst design. In addition, the effect of post-synthesis thermal treatment on CdS crystallinity and photocatalytic performance is examined using rGO-PEI@CdS as a representative system, providing insight into the balance between semiconductor crystallinity and interfacial chemical integrity.

## 2. Results

### 2.1. Covalent PEI Functionalization and Reduction of GO—Formation of rGO-PEI

The reaction between GO and PEI relies on the high density of oxygen-containing functional groups present on the GO surface, which act as reactive sites for the covalent incorporation of amine-rich polymeric species.

**Figure 2:**
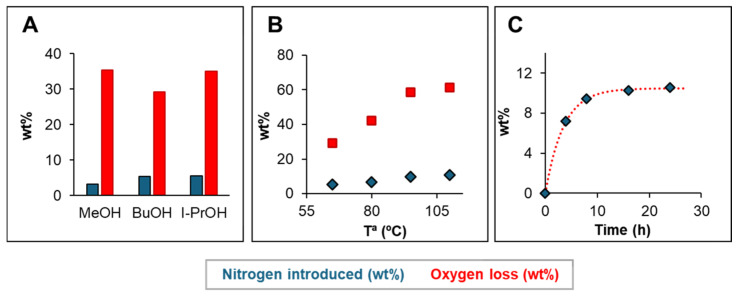
Influence of synthetic parameters on the covalent functionalization of GO with PEI. (**A**) Effect of solvent on nitrogen incorporation, and oxygen loss at 65 °C. (**B**) Effect of reaction temperature on nitrogen incorporation and oxygen loss using *n*-butanol as solvent. (**C**) Effect of reaction time on nitrogen incorporation at 110 °C, showing saturation behavior at longer reaction times. Nitrogen content was determined by combustion elemental analysis, while oxygen content was calculated by the difference from the remaining element composition.

The synthesis of the GO-PEI hybrid was optimized through a systematic evaluation of key synthetic parameters, including reaction time, solvent, and temperature. In order to assess the influence of the solvent, a series of GO-PEI hybrids were prepared in methanol, isopropanol, and *n*-butanol at 65 °C. The extent of PEI incorporation was quantified by combustion elemental analysis through the nitrogen content of the resulting materials (Figure 2). Comparable nitrogen mass fractions of approximately 5.5 wt% were obtained for *n*-butanol and isopropanol, whereas significantly lower values were observed when methanol was employed as the reaction medium. Among the solvents investigated, *n*-butanol was selected for further studies, as it allows operation at higher reaction temperatures, which is conducive to enhanced PEI incorporation.

Subsequently, the effect of reaction temperature was investigated using *n*-butanol as a solvent. A progressive increase in nitrogen incorporation was observed with increasing temperature, reaching a content of approximately 10.7 wt% at 110 °C. Finally, the influence of reaction time at this temperature was examined, revealing an exponential increase in nitrogen content at shorter reaction times followed by a saturation at longer durations. Based on these results, a reaction time of 24 h was selected, ensuring a high degree of GO functionalization with PEI.

Related with the increased incorporation of PEI, a pronounced decrease in the oxygen content of GO was observed, particularly at elevated reaction temperatures, reaching reductions of up to 60% at 110 °C. This observation suggests that, under the applied conditions, the presence of PEI and the associated thermal treatment induce partial reduction of GO during the functionalization process. As a result, the final material can be described as a reduced graphene oxide-polyethyleneimine hybrid (rGO-PEI). Similar surface reduction effects during polymer-assisted functionalization of GO have been previously reported in the literature [16,17]. On the basis of these optimized conditions, the rGO-PEI hybrid was synthesized following the methodology described in Section 3.2.1.

**Figure 3:**
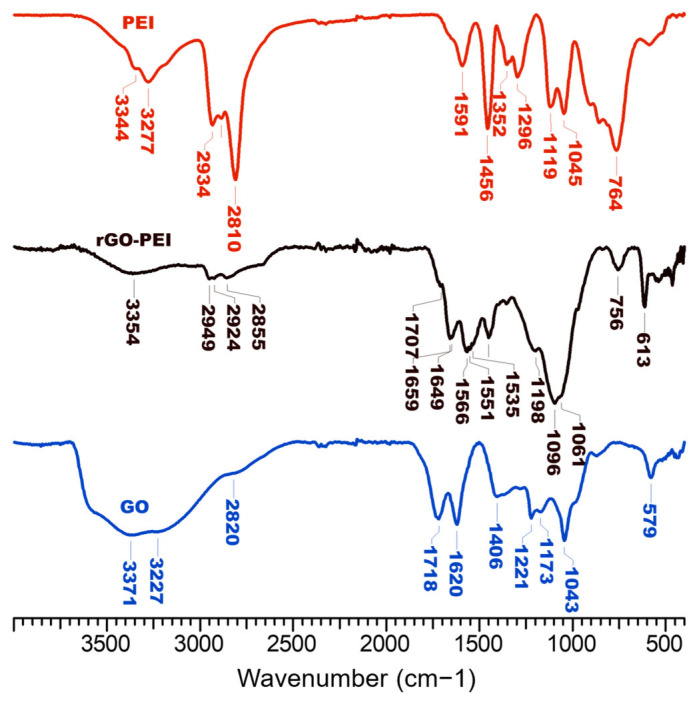
FTIR spectra of PEI-1.8K (red), GO (blue) and rGO-PEI (black).

Fourier-transform infrared (FTIR) spectroscopy was first employed to confirm the successful anchoring of PEI onto the GO surface. A direct comparison between the FTIR spectra of GO and the rGO-PEI hybrid prepared under optimized conditions (Figure 3) reveals the appearance of characteristic aliphatic -CH_2_- stretching vibrations at 2949, 2923, 2854, and 2821 cm^−1^, consistent with the FTIR fingerprint of PEI. In addition, the broad O-H stretching band of GO in the 3600–3300 cm^−1^ region undergoes a noticeable modification after functionalization, which can be attributed to the overlap between O-H and N-H stretching contributions after PEI incorporation.

Significant changes are also observed in the carbonyl and C-O regions. The intense C=O stretching band of GO centered at 1718 cm^−1^ significantly decreases in intensity in the rGO-PEI hybrid, while new bands emerge at 1659–1649 cm^−1^ and at 1566 cm^−1^, which can be assigned to amide-type vibrations and N-H bending/C-N stretching modes, respectively [18]. These features indicate chemical interactions between the amine groups of PEI and the oxygen-containing functionalities present on the GO surface. Additionally, the attenuation of the C-O stretching band at 1043 cm^−1^, together with the appearance of a broad band in the 1095–1061 cm^−1^ region, further supports the modification of C-O groups and the formation of C-N bonds. Collectively, these FTIR changes are consistent with PEI functionalization accompanied by a partial reduction of GO.

**Figure 4:**
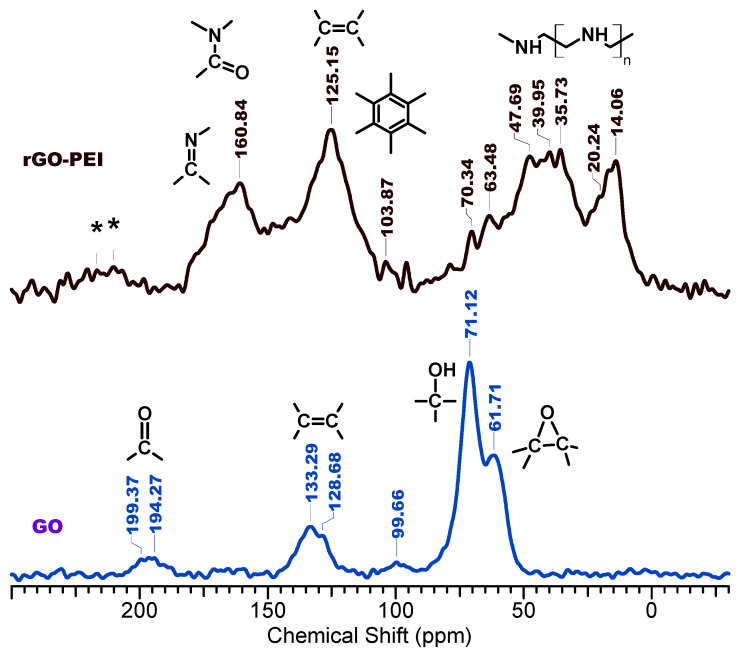
Solid-state ^13^C NMR spectra of GO (blue) and the rGO-PEI hybrid prepared at 110 °C (black), highlighting the distinct carbon environments and their corresponding chemical shifts. Asterisks (*) denote spinning sidebands.

Complementary structural information was obtained from solid-state ^13^C NMR spectroscopy (Figure 4). The spectrum of GO exhibits its characteristic resonances at approximately 60 ppm (epoxy C-O-C) and 70 ppm (C-OH), a broad sp^2^ carbon signal in the 125–135 ppm region (C=C), and carbonyl contributions at 194–199 ppm, consistent with previous reports on GO materials [19,20]. Upon PEI functionalization, the rGO-PEI hybrid displays new aliphatic resonances between 32 and 48 ppm, which can be attributed to the -CH_2_- groups of PEI, confirming polymer incorporation into the solid material. Additionally, an increase in intensity and broadening of the sp^2^ carbon signal around 125 ppm is observed. This behavior suggests an increased contribution of conjugated carbon domains relative to the starting GO. The appearance of a resonance at around 165 ppm, compatible with amide-type carbonyl carbons, further supports chemical interactions between the amine groups of PEI and oxygen-containing functionalities on the GO surface. Minor signals in the 14–35 ppm region, as well as around 60 ppm, are attributed to residual *n*-butanol adsorbed on the material [21]. Thereby, the relative decrease in oxygenated carbon signals (60–70 ppm) compared to sp^2^ carbon contributions corroborates the partial reduction inferred from FTIR analysis.

**Figure 5:**
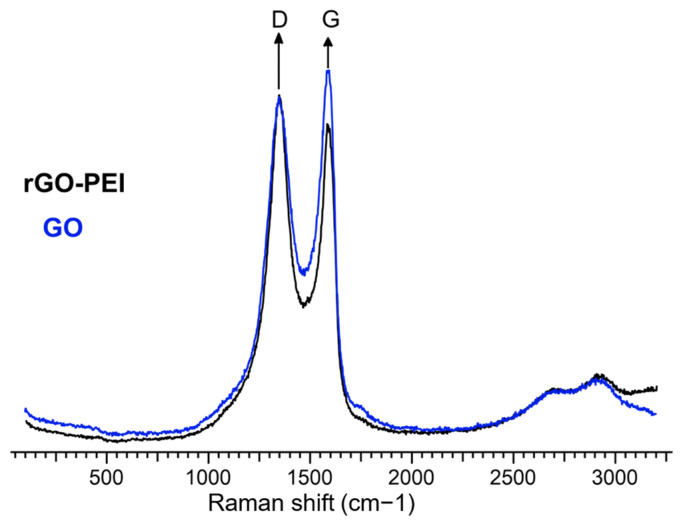
Raman spectrum acquired at 532 nm for GO (blue) and rGO-PEI (black).

Raman spectroscopy provides additional insight into the evolution of the carbon framework during PEI functionalization (Figure 5). Both GO and rGO-PEI exhibit the characteristic D and G bands at approximately 1350 cm^−1^ and 1597 cm^−1^, respectively, associated with defect-activated modes and in-plane vibration of sp^2^ hybridized carbon atoms. The rGO-PEI hybrid displays an increased I_D_/I_G_ ratio relative to the starting GO, a behavior commonly reported for partial rGO. This increase does not necessarily indicate enhanced oxidation but is instead attributed to the removal of oxygen-containing functional groups and the concomitant reorganization of the sp^2^ carbon network into smaller graphitic domains. This structural rearrangement leads to an increased relative density of edges and defect sites, which enhances the activation of the D band [22,23].

Taken together, the combined FTIR, solid-state ^13^C NMR, and Raman spectroscopic results demonstrate that PEI functionalization induces both chemical modification of the GO surface and partial reorganization of the carbon framework. The resulting rGO-PEI material can thus be described as an interfacial hybrid exhibiting a partially restored graphenic character, characterized by fragmented sp^2^ domains, a high density of edges and defects, and a surface enriched in amine functionalities. This structural and chemical description is fully consistent with the high nitrogen contents determined by elemental analysis.

### 2.2. PDI-Mediated Non-Covalent Assembly—Formation of PDI-PEI, G-Sheet-PDI-PEI and GNP-PDI-PEI

In contrast to GO, the surface functionalization of GNPs or G-sheets with PEI cannot be achieved through covalent pathways due to the low density of oxygen-containing functional groups on their surfaces.

**Figure 6:**
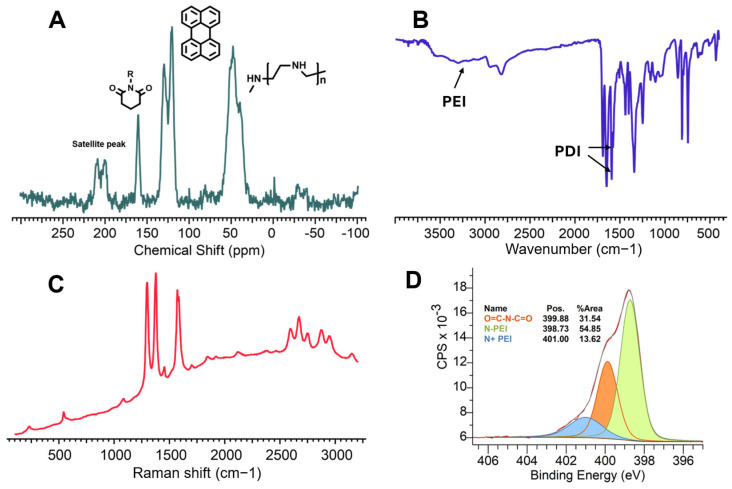
Spectral analysis of PDI-PEI: (**A**) solid-state ^13^C-NMR, (**B**) FTIR), (**C**) Raman spectrum at 532 nm and (**D**) high-resolution N1s XPS spectrum.

To achieve this, a non-covalent surface functionalization strategy was employed, based on perylenediimide derivatives obtained from the reaction of perylene-3,4,9,10-tetracarboxylic dianhydride (PDA) with PEI. In these PDI-PEI derivatives, the PDI aromatic fragment acts as a noncovalent anchoring moiety through π-π stacking interactions with the graphene surface, providing the noncovalent connection of the PEI fragment to the graphene-type surface. To gain insight into the structure and properties of the hybrid system, PDI-PEI material in the absence of graphene was synthesized as a model compound following a recent advanced procedure for the preparation of PDI derivatives [24]. The solid-state ^13^C-NMR spectrum of PDI-PEI (Figure 6A) exhibits the characteristic resonances of both components, including signals in the 30–60 ppm region corresponding to the aliphatic CH_2_ groups of PEI [25,26], aromatic carbon signals around 125 ppm assigned to the perylene core, and resonances at approximately 160 ppm attributed to the imide carbonyl carbons [27,28]. The complementary FTIR spectrum (Figure 6B) displays the characteristic vibrational features of PDI units, with intense absorption bands at approximately 1690 and 1646 cm^−1^, assigned to the asymmetric and symmetric stretching modes of the imide carbonyl (C=O) groups. These bands provide clear evidence for the successful imidization of the perylene precursor and the formation of PDI units [24,29]. In addition, multiple absorption bands in the 3000–2800 cm^−1^ region are observed and attributed to aliphatic C-H stretching vibrations of the -CH_2_- groups in PEI, further confirming the presence of the polymer in the hybrid. Also, the Raman spectrum of the functionalized material (Figure 6C) displays PDI-associated marker bands at 1306 and 1380 cm^−1^, corresponding to aromatic ring stretching modes and consistent with the reported Raman spectra of PDI derivatives [30,31]. Finally, the XPS survey spectrum (Figure S2) indicates a nitrogen content of 11.1 at%. The high-resolution N1s spectrum (Figure 6D) reveals the presence of different nitrogen species in the structure, including the amine nitrogen of PEI at 398.7 eV, protonated amine nitrogen of PEI at 401.0 eV, and imide nitrogen at 399.9 eV. The relative contribution of the imide nitrogen is approximately 32%, which is comparable to the expected fraction of primary amine groups in PEI (approx. 33%). This result is consistent with the previously reported reaction mechanism [24], in which imide formation occurs selectively at primary amine sites. The close agreement between these values further indicates a high degree of conversion of the available primary amines, while secondary and tertiary amines remain unreacted.

**Figure 7:**
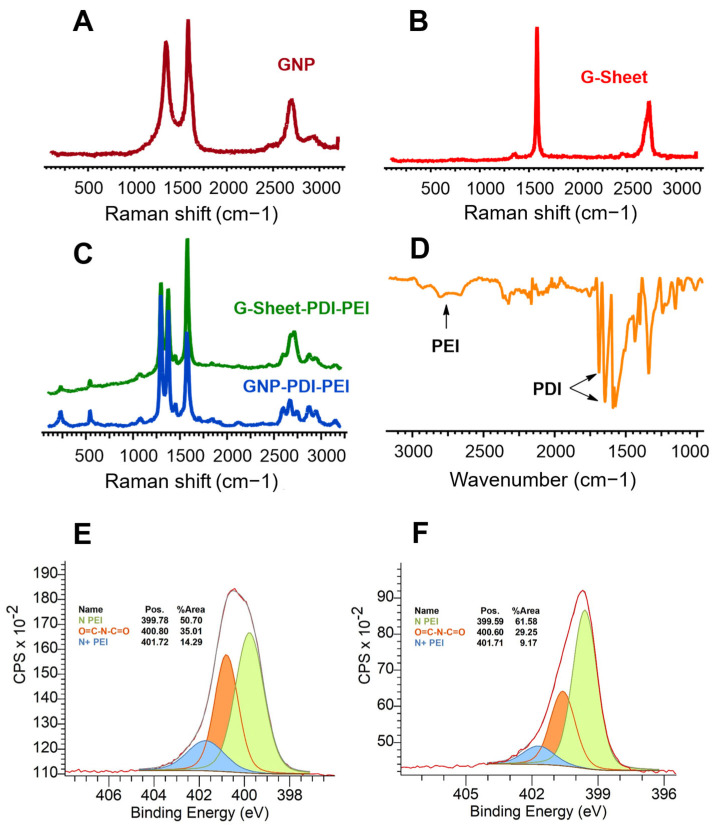
Raman spectra acquired at 532 nm of (**A**) GNP, (**B**) G-Sheet, (**C**) GNP-PDI-PEI and G-Sheet-PDI-PEI. (**D**) FTIR spectrum of GNP-PDI-PEI and high-resolution N1s spectra of (**E**) G-Sheet-PDI-PEI and (**F**) GNP-PDI-PEI.

The noncovalent assembly of PDI-PEI-functionalized graphene hybrids was carried out in a one-pot procedure involving the in situ formation of perylenediimide (PDI) derivatives and their concomitant anchorage by π-π stacking on the graphene surface (Section 3.2.2). The characterization of the Graphene-PDI-PEI materials was based on spectroscopic features of the graphene-type precursors and the structural insights obtained from the model PDI-PEI system. Raman spectroscopy was first employed to examine the structural features of the pristine graphene supports. As shown in Figure 7A, GNP exhibits a pronounced D band, consistent with a high density of edges and structural defects. In contrast, the G-Sheet (Figure 7B) displays an intense and narrow G band, a weak D band, and a well-defined 2D band, indicative of a lower defect density and more extended sp^2^ domains. These intrinsic differences are preserved after functionalization, as the characteristic Raman spectra of GNP-PDI-PEI and G-Sheet-PDI-PEI (Figure 7C) retain the characteristic G and 2D bands, indicating that the noncovalent assembly process preserves the integrity of the carbon lattice. In addition, the Raman spectra also exhibit the characteristic PDI-related bands, in line with the signatures previously identified in the model system PDI-PEI (Figure 6C). Notably, a very low fluorescence background is observed despite the presence of PDI units. Considering the intrinsically strong fluorescence of PDI derivatives, this pronounced fluorescence quenching can be attributed to intimate π-π interactions between PDI and the graphene surface [32,33]. Complementary FTIR analysis (Figure 7D) further supports the formation of the hybrid architecture, reproducing the key vibrational features of the PDI-PEI model system (Figure 6B), indicative of the effective incorporation of the PDI-PEI organic component onto the graphene surface.

In addition, high-resolution N 1s XPS spectra provide further insight into the chemical environment of the PDI-PEI layer after immobilization on the graphenic supports (Figure 7E,F). In both G-Sheet-PDI-PEI and GNP-PDI-PEI, the deconvolution reveals three contributions assigned to neutral amine nitrogen from PEI, imide nitrogen, and protonated amine species, indicating that the main nitrogen environments identified in the PDI-PEI model system are retained after immobilization on the graphene surfaces (Figure 6D). This result indicates that the interfacial layer formed on graphene retains the essential chemical features of the PDI-PEI model. Although the same nitrogen environments are observed in both hybrids, their relative contributions differ depending on the graphenic substrate. G-Sheet-PDI-PEI exhibits a slightly higher fraction of imide nitrogen (35.0%) together with a larger contribution of protonated amine species (14.3%), whereas GNP-PDI-PEI shows a lower imide content (29.3%) and a markedly higher proportion of neutral amine nitrogen (61.6%). These differences can be attributed to variations in the relative amount of PEI incorporated during the functionalization process, with GNP-PDI-PEI containing a higher proportion of PEI-derived amine functionalities. Overall, these findings demonstrate the formation of well-defined PDI–PEI interfaces on graphene-based supports and highlight the influence of the graphenic substrate on the relative contributions of PEI and PDI nitrogen species.

### 2.3. PEI-Mediated CdS Formation—Preparation of rGO-PEI@CdS, GNP-PDI-PEI@CdS, G-Sheet-PDI-PEI@CdS and rGO-PEI@CdS-500

The CdS formation process was carried out through the repetitive alternating addition of small aliquots of Cd^2+^ and S^2−^ precursors at identical concentrations. The number of addition cycles was programmed to enable a gradual increase in CdS content, targeting a final composite composition of approximately 95 wt% CdS and 5 wt% graphenic material. The ratios between graphene-PEI precursor and amount of ion (Cd^2+^ or S^2−^) in the aliquots were defined based on the nitrogen content of each hybrid material, which was used as a chemical descriptor of the capacity of the hybrid graphene-PEI derivative to complex Cd^2+^ ions. The nitrogen content of the PEI hybrids was determined by combustion elemental analysis (Table 1). In parallel, XPS analysis was performed to probe the surface composition (Table 2). Since PEI functionalization is inherently connected to the hybrid surface, the comparison between both techniques is better interpreted in terms of the effective exposure of nitrogen sites relative to the carbon support. In this regard, rGO-PEI and GNP-PDI-PEI show close agreement between bulk- and surface-normalized N/C ratios (Table S1), with values of 0.15 and 0.07, indicating that the XPS-probed surface is representative of the overall accessible composition. In contrast, G-Sheet-PDI-PEI exhibits a pronounced discrepancy, with atomic N/C ratios of 0.0036 (derived from elemental analysis) and 0.042 (XPS), corresponding to an approximately twelvefold increase at the surface relative to the bulk composition. This indicates that despite its low overall nitrogen content, the PEI functionalities are preferentially concentrated at the most accessible outer regions of the support, where they remain accessible for active Cd^2+^ coordination.

Therefore, based on the nitrogen content determined by combustion elemental analysis, a fixed molar ratio of 12 mol N per mol Cd was applied for rGO-PEI@CdS and GNP-PDI-PEI@CdS to ensure comparable coordination conditions. Accordingly, the concentrations of CdCl_2_·2.5H_2_O and the Na_2_S precursor were adjusted to 63.7 mM and 37.3 mM, resulting in a total of 207 and 353 addition cycles, respectively. In contrast, the significantly lower nitrogen content of G-Sheet-PDI-PEI (0.4 wt%) prevents the direct application of the same stoichiometric conditions. Maintaining the 12 mol N/mol Cd ratio would require dilute precursor solutions (2.5 mM) and an impractically large number of addition cycles (approx. 5270), corresponding to an estimated reaction time of several weeks. However, the surface enrichment revealed by XPS indicates that the available nitrogen functionalities remain effectively exposed, providing accessible coordination sites for Cd^2+^ despite the low overall nitrogen content. Therefore, to overcome this limitation, the precursor concentration was increased to 25.3 mM, reducing the number of addition cycles to 519, while still enabling a CdS loading comparable to that of the other materials.

**Figure 8:**
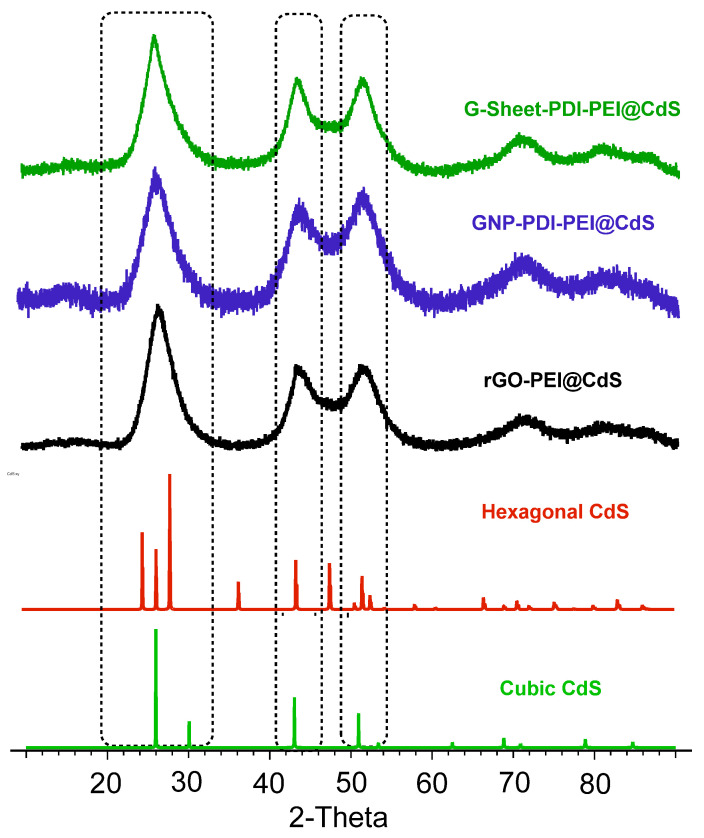
XRD patterns of different CdS-based hybrids: G-Sheet-PDI-PEI@CdS (green), GNP-PDI-PEI@CdS (blue) and rGO-PEI@CdS (black), together with the simulated pattern of cubic (zincblende) and hexagonal (wurtzite) CdS used as reference for phase identification.

XRD patterns (Figure 8) of rGO-PEI@CdS, GNP-PDI-PEI@CdS, and G-Sheet-PDI-PEI@CdS exhibit the same set of diffraction maxima at comparable 2θ positions, indicating that CdS is formed reproducibly across all graphene supports under the PEI-mediated growth protocol. The most intense reflections (26.5°, 44° and 52°) are consistent with nanocrystalline CdS, whose low crystallinity precludes a clear distinction between the hexagonal and cubic phases. The broad reflections indicate small coherent crystallite domains, consistent with restricted CdS crystal growth under the PEI-mediated nucleation environment. Notably, the close similarity of the diffraction profiles across all materials indicates that the final CdS crystalline is not strongly altered by the nature of the underlying carbon scaffold. This suggests that while PEI-derived functionalities provide the coordination sites for Cd^2+^ species, the overall CdS formation process is primarily governed by the PEI-assisted sequential precursor addition protocol, leading to comparable crystalline CdS domains across all systems.

**Figure 9:**
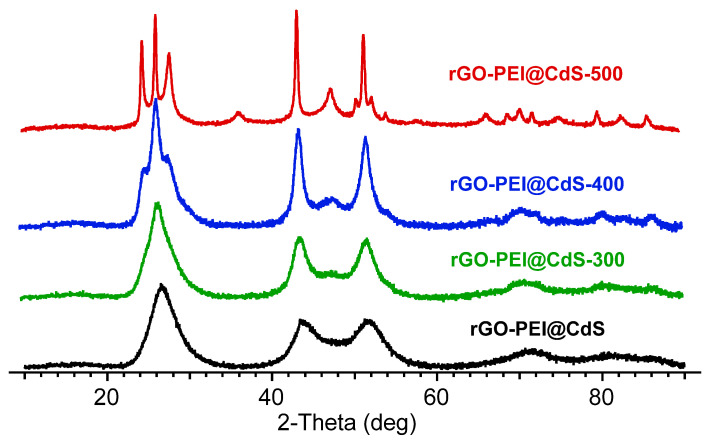
XRD patterns of the rGO-PEI@CdS hybrid recorded at different temperatures: room temperature (black), 300 °C (green), 400 °C (blue) and 500 °C (red).

In addition, to evaluate the effect of thermal treatment on the crystallinity of CdS, the rGO-PEI@CdS hybrid was subjected to controlled heating. The XRD patterns recorded after thermal treatment at 300, 400 and 500 °C are summarized in Figure 9.

The XRD pattern of the prepared rGO-PEI@CdS hybrid exhibits broad diffraction features characteristic of nanocrystalline CdS, with limited domain size. Upon thermal treatment at 300 °C, a moderate increase in the sharpening and intensity of the diffraction peaks is observed, indicating the onset of crystallite growth while preserving the same CdS phase. Further heating to 400 and 500 °C results in progressively sharper and more intense reflections, evidencing enhanced crystallinity and growth of CdS domains, which can now be identified as hexagonal (wurtzite) [34,35]. Overall, these results indicate that the initial PEI-mediated growth yields structurally well-defined CdS nanocrystals, whose crystallinity can be tuned by post-synthetic thermal treatment.

SEM analysis was performed to evaluate the morphology of the CdS-based hybrid materials and to assess the spatial relationship between CdS and the graphene-derived supports.

**Figure 10:**
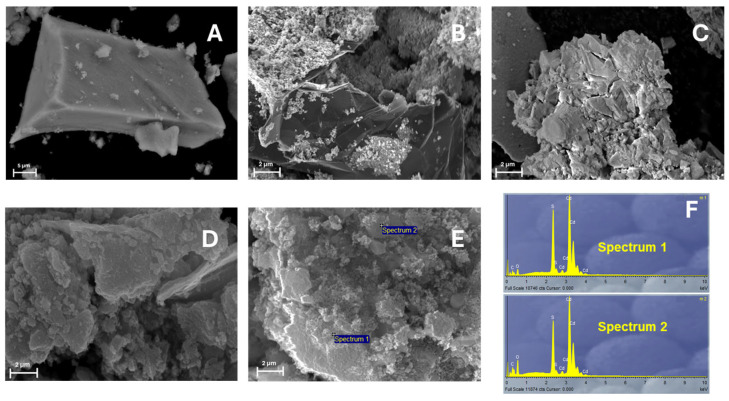
SEM images of the CdS-based materials: (**A**) bare CdS, (**B**) G-Sheet-PDI-PEI@CdS, (**C**) GNP-PDI-PEI@CdS, (**D**) rGO-PEI@CdS, (**E**) rGO-PEI@CdS-500, and (**F**) EDX spectra collected from the selected regions marked in panel (**E**), showing intense Cd and S signals together with minor C and O contributions.

Bare CdS synthesized in the absence of a graphene-derived support (Figure 10A) exhibits a morphology dominated by compact, bulk-like aggregates. In contrast, the CdS/graphene-derived hybrids display markedly different morphologies, indicating that the presence of the functionalized carbon phase influences the spatial organization of CdS during formation. In G-Sheet-PDI-PEI@CdS (Figure 10B), sheet-like regions associated with G-Sheet remain visible, with irregular CdS-rich granular deposits distributed over their surface, supporting CdS formation in close spatial association with the PDI-PEI-functionalized graphene surface. GNP-PDI-PEI@CdS (Figure 10C) displays a similar CdS morphology, characterized by CdS-rich deposits distributed over stacked and fragmented graphenic-derived domains. Therefore, although the underlying carbon supports are different, both G-Sheet-PDI-PEI@CdS and GNP-PDI-PEI@CdS exhibit CdS-rich deposits, mainly as granular domains associated with the graphenic phase. By contrast, rGO-PEI@CdS (Figure 10D) displays a more distinct morphology, in which CdS-rich domains appear as more continuous, clearly layered regions associated with rGO, suggesting that CdS formation was deeply influenced by the sheet morphology of the rGO support. Thus, unlike unsupported CdS, which forms compact bulk-like aggregates, the hybrids are characterized by CdS-rich domains spatially associated with the graphene-derived phase.

After thermal treatment at 500 °C, rGO-PEI@CdS-500 (Figure 10E) retained the layered morphology of its precursor, while the CdS phase gained considerable crystallinity, according to its XRD pattern. The latter also confirms the plate-like morphology of the CdS crystals through the decreased intensity and broadening of the wurtzite-type reflexions, with Miller indices belonging to the (1 0 X) family, which account for platelet-type crystals with the shortest dimension along the [1 0 0] direction (Figure S3). Moreover, EDX spectra collected from selected regions of rGO-PEI@CdS-500 (Figure 10F) show intense Cd and S signals, indicating that the analyzed sheet-like features contain a substantial CdS contribution and cannot be assigned exclusively to the graphenic phase.

Finally, the optical band gap of the hybrid materials was estimated from Tauc plots [36,37] following the intersection method approach [38] (Figure S4), yielding values of 2.45, 2.44, and 2.48 eV for rGO-PEI@CdS, G-Sheet-PDI-PEI@CdS, and GNP-PDI-PEI@CdS, respectively. The similarity of these values indicates that the CdS domains formed through the PEI-mediated growth protocol exhibit comparable optical responses, despite the distinct nature of the graphenic support. In contrast, the thermally treated sample rGO-PEI@CdS-500 shows a reduced band gap of 2.32 eV, which is consistent with the increased crystallinity and domain growth observed by XRD. Overall, these results demonstrate that the PEI-mediated assembly strategy leads to CdS-based hybrids with closely related band gap energies, while post-synthetic thermal treatment provides an additional route to modulate the optical properties of the CdS phase.

### 2.4. Photocatalytic Studies

**Figure 11:**
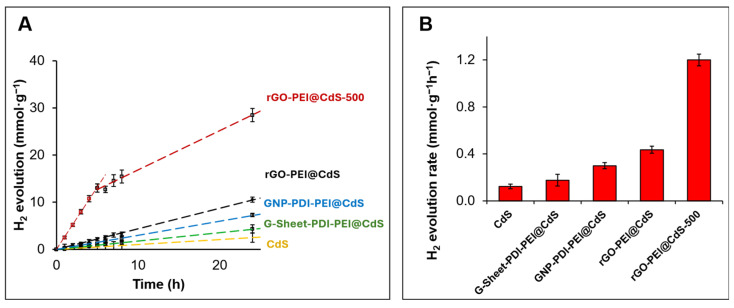
(**A**) Photocatalytic hydrogen evolution profiles obtained with 5 mg of the graphene-CdS catalysts in an aqueous Na_2_S and Na_2_SO_3_ sacrificial system (0.35M, 0.25M) under visible light reduction and (**B**) comparison of the average H_2_ evolution rates of the different graphene-PEI@CdS samples. Error bars represent mean ± s.d. of three independent experiments.

Photocatalytic hydrogen evolution experiments were carried out using a catalyst loading of 1.0 mg·mL^−1^ and without the presence of any noble-metal cocatalysts. The time-dependent hydrogen production profiles and the corresponding average H_2_ production are summarized in Figure 11.

The hydrogen evolution measurements reveal a clear dependence of photocatalytic performance on the nature of the graphene-derived support. Among the evaluated materials, rGO-PEI@CdS exhibits the highest hydrogen production rate, reaching 0.44 mmol g^−1^ h^−1^. In comparison, GNP-PDI-PEI@CdS and G-Sheet-PDI-PEI@CdS display lower activities of 0.30 and 0.18 mmol g^−1^ h^−1^, respectively. These materials were tested under simulated solar irradiation (AM1.5G, 1 sun) and in the absence of an external noble metal cocatalyst, yielding solar-to-chemical conversion efficiencies (SCCs) of 0.018, 0.012 and 0.007%, respectively, considering that H_2_ evolution is coupled to oxidation of the sacrificial redox system, Na_2_S/Na_2_SO_3_, rather than to O_2_ evolution [39] (see Supplementary Materials). Notably, all hybrid materials exhibit higher hydrogen evolution rates than bare CdS prepared under similar conditions (0.12 mmol g^−1^ h^−1^), in agreement with the value previously reported by our group [40].

**Figure 12:**
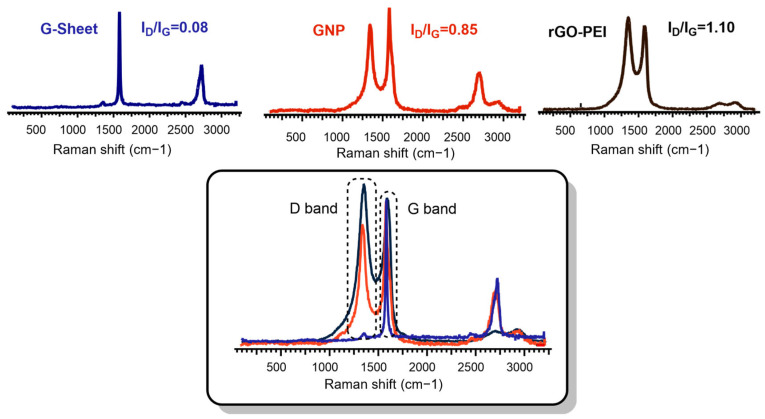
Raman spectra of graphene-based materials showing increasing I_D_/I_G_ ratios from G-Sheet to rGO. Spectra are normalized to the G band (1580 cm^−1^); inset highlights the D-G region.

These differences are unlikely to arise primarily from changes in the CdS crystalline phase, as XRD analysis confirms comparable nanocrystalline CdS across all hybrids. Instead, the observed trend suggests that the graphene-derived support plays an important role in modulating the photocatalytic performance of the CdS/carbon hybrids. A first approach to the structural analysis of the graphenic supports can be obtained by Raman spectroscopy. Under identical Raman conditions (532 nm), rGO exhibits the highest I_D_/I_G_ (=1.10; Figure 12, black), followed by GNP (=0.85; Figure 12, red), whereas the G-Sheet shows a markedly lower value (=0.08; Figure 12, blue). This sequence reflects a progressive decrease in defect-related contributions and a higher degree of ordered sp^2^ domains from rGO to G-Sheet. Therefore, the higher activity of rGO-PEI@CdS is consistent with the presence of a more defect-rich carbon framework, which may provide additional interfacial sites for interaction with CdS. As discussed above, rGO-PEI@CdS exhibits a more evident sheet-like CdS morphology than the G-Sheet- and GNP-based hybrids, suggesting a larger apparent CdS/carbon interfacial area. Therefore, the enhanced activity of rGO-PEI@CdS is likely associated with the combined effect of several factors: a higher defect-related Raman contribution, a more pronounced CdS-rich sheet-like morphology and a more favorable CdS/carbon interfacial arrangement.

The photocatalytic hydrogen evolution performance of the thermally treated rGO-PEI@CdS-500 sample was additionally evaluated to assess the impact of increased CdS crystallinity on catalytic activity. Compared to the as-prepared rGO-PEI@CdS hybrid, the sample treated at 500 °C exhibits a higher initial hydrogen evolution rate, consistent with the enhanced crystallinity of CdS observed by XRD. Over the first 24 h of irradiation, rGO-PEI@CdS-500 reached an overall average hydrogen production rate of 1.20 mmol g^−1^∙h^−1^. However, the analysis of the production profile revealed a clear change in the hydrogen evolution rate with irradiation time. During the first 5 h, the material displayed a high production rate of 2.50 mmol g^−1^ h^−1^, whereas this value decreased to 0.83 mmol g^−1^∙h^−1^ during the subsequent 5–24 h interval. In line with this non-linear behavior, the SCC was calculated for the 0–5 h and 5–24 h intervals (see Supplementary Materials), yielding an SCC (for 5 h) of 0.100% and SCC (5–24 h) of 0.033%. The decrease in hydrogen evolution rate after approximately 6 h indicates partial activity loss during prolonged irradiation. This behavior contrasts with the more stable performance of the non-thermally treated hybrid and suggests progressive deactivation processes. A plausible explanation is that the thermal treatment at 500 °C substantially modifies the PEI-derived interfacial environment. Thermogravimetric analysis of PEI (Figure S6) shows a major mass-loss below 400–450 °C, with almost no residual mass at higher temperatures, indicating that treatment at 500 °C can severely compromise the integrity of the amine-rich PEI component. As a result, the PEI-mediated coordination environment linking CdS and rGO may be partially altered, decreasing the effectiveness of interfacial charge separation and long-term photocatalytic stabilization.

Additional evidence of material alteration is provided by optical microscopy and Raman analysis of rGO-PEI@CdS-500 before and after 24 h of hydrogen evolution (Figure S5). After the photocatalytic test, the catalyst was recovered by filtration and dried at 120 °C for 2 h prior to analysis. The recovered material (Figure S5D,E) exhibits a markedly darker and more irregular appearance compared with the pristine thermally treated sample (Figure S5A,B). In parallel, representative Raman spectra collected after photocatalysis (Figure S5F) show a pronounced increase in the spectral background and less-resolved vibrational features, in contrast to the clearer CdS-related Raman response observed before irradiation (Figure S5C). These observations do not allow a single deactivation pathway to be assigned, but they support the occurrence of surface or interfacial modification during prolonged photocatalytic operation.

Overall, these results highlight the critical balance between CdS crystallinity and interfacial chemical integrity, demonstrating that while thermal treatment enhances short-term activity, the preservation of PEI-mediated interfacial functionalities is essential for sustained photocatalytic hydrogen production.

## 3. Materials and Methods

### 3.1. Materials

Graphene oxide (GO) was supplied by Nanoamor (16840, Houston, TX, USA). According to the supplier specifications, GO consists of fewer than 10 layers, with lateral dimensions in the range of 0.5–3.0 μm, a thickness of 0.5–1.2 nm, and a stated purity above 99%. Graphene nanoplatelets (GNP) were purchased from Nanografi Nano Technology (06531, Çankaya/Ankara, Turkey). The material is characterized by an average lateral size of 1.5 μm diameter, a thickness of 3 nm and a purity >99%. Low-defect graphene sheets (G-Sheets) were also supplied by Nanografi Nano Technology (06531, Çankaya/Ankara, Turkey). According to the manufacturer, this material exhibits a lateral dimension of up to 35 μm. Hyperbranched polyethyleneimine (PEI, M_n_ 1800 g/mol, pure grade) and sodium sulfide hydrate (Na_2_SxH_2_O, 59–65%) were provided by Merck KGaA (64293 Darmstadt, Germany). Perylene-3,4,9,10-tetracarboxylic dianhydride (PDA, 98%) was provided by Indagoo Research Chemical. Cadmium chloride hemipentahydrate (CdCl_2_·2.5H_2_O, 99%) and high-purity *n*-butanol, isopropanol and methanol (HPLC gradient quality) were supplied by Panreac Química (08211, Castellar del Vallès, Spain). Tert-butylamine (98%) was purchased from Thermo Fisher Scientific (02451, Waltham, MA, USA). To obtain the anhydrous compound, it was magnetically stirred over potassium hydroxide (KOH) pellets for 15 h to remove residual moisture. The dried amine was then transferred to a vial containing activated molecular sieves of 4Å and stored until use.

The total contents of nitrogen, carbon and hydrogen in rGO-PEI and GNP-PDI-PEI were determined by combustion elemental analysis using a LECO TruSpec Micro analyzer (Leco Corporation, St. Joseph, MI, USA) operating at 1100 °C. The solid-state NMR spectra of the rGO-PEI hybrids were recorded on a Bruker Avance 400 spectrometer (Bruker Corporation, Billerica, MA, USA) equipped with a 9.4 T superconducting magnet with a standard hole (nominal operating frequencies of 400 MHz for ^1^H and 100 MHz for ^13^C). ^13^C cross-polarization magic-angle spinning (CP/MAS) experiments were performed with proton decoupling, using a contact time of 1 ms and a recycle delay (D1) of 0.5 s. The spinning speed was fixed at 11 kHz with a total acquisition time per scan of 0.0399 s. Fourier-transform infrared (FTIR) spectra were recorded in the 400–3200 cm^−1^ range using a Bruker Vertex 70 spectrometer (Bruker Corporation, Billerica, MA, USA). The measurements were performed with a Platinum ATR accessory with pressure control and an air-cooled DLaTGS detector with a KBr window for mid-infrared region. Raman spectra were acquired using a confocal Raman microscope (Bruker Corporation, Billerica, MA, USA) with a Peltier-cooled Renishaw Centrus CCD detector with 1024 × 256 pixels. A laser operating at a wavelength of 532 nm with a maximum output power of 50 mW was used as the illumination source in the Raman analyses. X-ray diffraction (XRD) patterns were recorded on a Panalytical Empyrean diffractometer (Almelo, The Netherlands) using Cu Kα radiation (λ = 1.5406 Å) in the 2θ range from 5° to 90°. Data were collected using a step size of 0.013 and a counting time of 68.595 s per step. X-Ray Photoelectron Spectroscopy (XPS) was employed to determine the surface nitrogen content. Measurements were performed using a Kratos model INA-X SPECS apparatus (Kratos Analytical, Shimadzu Corporation, Kyoto, Japan) equipped with a double anode X-ray source (Mg/Al) with a power of 450 W and a hemispherical electron analyzer connected to a delay-line detector (DLD).

### 3.2. Synthetic Procedures

#### 3.2.1. Covalent PEI Functionalization—Formation of rGO-PEI

GO (200 mg) was dispersed in *n*-butanol (150 mL) and subjected to ultrasonic treatment for 15 min to obtain a homogeneous suspension. Subsequently, PEI (M_n_ = 1800 g/mol, 200 mg), previously dissolved in *n*-butanol (50 mL), was added to the GO dispersion. The resulting mixture was further sonicated for 6 min and then heated at 110 °C under an argon atmosphere for 24 h. After completion of the reaction, the system was allowed to cool to room temperature. The resulting solid was recovered by filtration and washed three times with methanol (3 × 15 mL) to remove unreacted species and residual solvent. Finally, the obtained rGO-PEI hybrid (170 mg) was dried using a drying gun at 65 °C for 4 h.

#### 3.2.2. PDI-Mediated Noncovalent Assembly—Formation of GNP-PDI-PEI and G-Sheet-PDI-PEI

For the preparation of the PDI-PEI model, perylenetetracarboxylic dianhydride (PDA, 200 mg) was first sonicated with anhydrous tert-butylamine (3 mL) in methanol (60 mL) until complete dissolution. This procedure, previously developed by our group [24], promotes efficient dissolution and activation of the dianhydride precursor, facilitating its subsequent reaction with amine-containing species to form PDI units. The resulting solution was then transferred into a flask containing PEI (Mn = 1800 g/mol, 130 mg) in methanol (30 mL). The reaction mixture was maintained under reflux with continuous stirring under an argon atmosphere for 15 h. After completion of the reaction, the solid obtained was filtered, washed with deionized water (2 × 20 mL) and subsequently dried using a drying gun at 90 °C for 2 h, yielding 294 mg of a red solid corresponding to the PDI-PEI hybrid.

The noncovalent assembly of PDI-PEI-functionalized graphene hybrids was carried out using a one-pot procedure though formation of perylenediimide (PDI) derivatives with concomitant anchorage by π-π stacking on the graphene surface. For the preparation of the GNP-PDI-PEI hybrid, perylenetetracarboxylic dianhydride (PDA, 10 mg) was first sonicated with anhydrous tert-butylamine (1.5 mL) in methanol (30 mL) until complete dissolution. The resulting solution was then transferred into a flask containing a dispersion of GNP (250 mg) and PEI (M_n_ = 1800 g/mol, 75 mg) in methanol (30 mL). The reaction mixture was maintained under reflux with continuous stirring under an argon atmosphere for 15 h, enabling the reaction between PDA and the amine groups of PEI to form PDI units, which subsequently anchored onto the graphene surface via noncovalent π-π interactions. After completion of the reaction, the solvent was slowly removed using a rotary evaporator at 300 mbar for 1 h, followed by full vacuum for 30 min to ensure complete solvent removal. The resulting solid was washed with deionized water (2 × 20 mL) and subsequently dried using a drying gun at 90 °C for 2 h, yielding 402 mg of a black solid corresponding to the GNP-PDI-PEI hybrid.

The G-Sheet-PDI-PEI hybrid was prepared following an analogous procedure. PDA (2.5 mg) was sonicated with anhydrous tert-butylamine (0.38 mL) in methanol (7.5 mL) until complete dissolution. The resulting solution was then transferred into a flask containing a sample of G-Sheet (80 mg), and the mixture was sonicated using a Bandelin Sonoplus ultrasonic processor operated to 20% amplitude in pulse mode (30% duty cycle) for 4 h until homogeneous dispersion was obtained. Subsequently, this dispersion was transferred to a flask containing PEI (M_n_ = 1800 g/mol, 23 mg) dissolved in methanol (7.5 mL). The reaction mixture was maintained under reflux with continuous stirring under an argon atmosphere for 15 h. The PDA loading was adjusted accordingly to achieve comparable functionalization of the graphene derivatives. After completion of the reaction, the solvent was slowly removed using a rotary evaporator at 300 mbar for 1 h, followed by full vacuum for 30 min to ensure complete solvent removal. After solvent removal, the solid was washed with deionized water (2 × 5 mL) and subsequently dried using a drying gun at 90 °C for 2 h, yielding 79 mg of a black solid corresponding to the G-Sheet-PDI-PEI hybrid.

#### 3.2.3. PEI-Mediated CdS Formation—Preparation of rGO-PEI@CdS, rGO-PEI@CdS-500, GNP-PDI-PEI@CdS and G-Sheet-PDI-PEI@CdS

CdS formation was carried out following a methodology previously developed within our research group [40,41], based on successive and stoichiometrically controlled precursor additions. In all cases, the same stepwise protocol was applied, while the total amount of Cd^2+^ and S^2−^ introduced was scaled according to the nitrogen content of each hybrid material. In a typical procedure, successive alternate additions of CdCl_2_·2.5H_2_O and Na_2_S solutions were performed, onto a dispersion of the graphene-based hybrid (10 mg of GNP-PDI-PEI, G-Sheet-PDI-PEI, or rGO-PEI) in deionized water (200 mL) at room temperature. In the specific case of G-Sheet-PDI-PEI, methanol was used as the dispersion medium instead of water due to the highly hydrophobic nature of the material, which prevents stable aqueous dispersion. The precursor concentrations were adjusted to 63.7 mM, 37.3 mM, and 25.3 mM for rGO-PEI@CdS, GNP-PDI-PEI@CdS and G-Sheet-PDI-PEI@CdS, respectively. In all cases, Cd^2+^ and S^2−^ precursor solutions were used at identical concentrations to ensure a 1:1 stoichiometric ratio during CdS formation. Accordingly, a total of 207, 353 and 519 addition cycles were applied, each consisting of the sequential addition of 0.1 mL of the Cd^2+^ and S^2−^ precursor solutions, with a fixed interval of 300 s between consecutive additions. The procedure was designed to obtain a final material composition containing approximately 95 wt% CdS and 5 wt% graphene-based components. After completion of the addition cycles, the resulting solid was recovered by filtration and thoroughly washed with deionized water (3 × 20 mL) to remove residual ions and unreacted species. In the case of the G-Sheet-based hybrid, the solid was washed with methanol instead of water. Finally, the materials were subsequently dried using a drying gun at 90 °C for 4 h. The procedure yielded green solid powders corresponding to rGO-PEI@CdS (195 mg), GNP-PDI-PEI@CdS (195 mg) and G-Sheet-PDI-PEI@CdS (197 mg). For comparison, a bare CdS reference sample was also prepared in the absence of both graphene-derived support and PEI, following the same precursor-addition conditions used for rGO-PEI@CdS: 63.7 mM CdCl_2_·2.5H_2_O and Na_2_S solutions, 207 addition cycles, 0.1 mL per precursor addition, and a fixed interval of 300 s between consecutive additions.

To assess the influence of thermal treatment on the crystallization behavior of CdS, the rGO-PEI@CdS hybrid was subjected to in situ heating during XRD measurements. The sample was heated up to 500 °C, and the corresponding XRD patterns were recorded to monitor changes in crystallinity and phase evolution.

#### 3.2.4. Photocatalytic Experiments

Photocatalytic hydrogen production experiments were conducted at 298.1 K and atmospheric pressure in a 140 mL doubled-walled flask sealed with a silicone rubber septum. A solar simulator (PICO G2V Optics Inc, Edmonton, AB, Canada) equipped with a standard AM1.5 G light source was used to provide simulated solar irradiation and initiate the photocatalytic reaction. The reactor was placed 1 cm from the light source, corresponding to 1 sun irradiation under AM1.5 G conditions, equivalent to 100 mW·cm^−2^. No additional UV or visible cut-off filters were used, since the experiments were designed to evaluate the photocatalysts under full simulated solar irradiation, resembling direct solar exposure conditions.

In a typical experiment, 5 mg of the photocatalyst (rGO-PEI@CdS, rGO-PEI@CdS-500, GNP-PDI-PEI@CdS and G-Sheet-PDI-PEI@CdS) was dispersed in 5 mL of an aqueous solution containing 0.35 M Na_2_S and 0.25 M Na_2_SO_3_ as sacrificial agents. A continuous magnetic stirrer was applied to maintain the photocatalyst’s uniform suspension throughout the experiment. Prior to irradiation, the system was purged with argon for 40 min to remove the dissolved oxygen. In each experiment the suspension was irradiated for 24 h; during this time, 0.02 mL of the generated gas was collected through the septum for analysis, at predesigned time intervals. The hydrogen content in the collected gas samples was quantified by gas chromatography (Agilent Technologies 7820A) with a thermal conductivity detector TCD, using argon as the carrier gas and a 5 Å molecular sieve column. Each photocatalytic experiment was repeated three times under identical conditions, and the results are reported as mean ± standard deviation.

## 4. Conclusions

In this study, an adaptable PEI-mediated interfacial assembly strategy was developed to address the long-standing challenge of achieving intimate contact between CdS and graphene-type co-catalysts in order to favor electronic transference between components and improve performance in CdS-graphene photocatalysts. By employing hyperbranched polyethyleneimine (PEI) as a central chemical mediator, CdS nucleation and growth were effectively controlled through Cd^2+^ coordination in the close vicinity of the graphenic surface, resulting in the reproducible formation of nanocrystalline CdS across all hybrid materials. This approach decouples the formation of the semiconductor phase from the intrinsic chemistry of the carbon support, enabling meaningful comparisons of graphene derivatives under similar synthetics.

The systematic evaluation of G-Sheet-PDI-PEI@CdS, GNP-PDI-PEI@CdS, and rGO-PEI@CdS hybrids reveals that photocatalytic hydrogen evolution is governed primarily by interfacial charge management rather than by differences in CdS crystallinity or phase. Among the studied systems, rGO-PEI@CdS displays the highest activity, which is attributed to the higher density of surface defects and residual oxygen-containing functionalities in rGO that facilitate electron trapping, suppress recombination, and promote efficient charge transfer to proton reduction sites. Importantly, these performances are achieved in the absence of noble-metal cocatalysts, underscoring the effectiveness of the PEI-mediated interfacial design.

Thermal treatment of rGO-PEI@CdS at 500 °C further demonstrates that increasing CdS crystallinity can enhance short-term photocatalytic activity, yielding an average hydrogen evolution rate of 1.20 mmol g^−1^ h^−1^ over 24 h. However, the observed loss of activity at extended reaction times highlights a critical trade-off between semiconductor crystallinity and interfacial chemical stability, as partial degradation of PEI-derived amine functionalities compromises sustained charge extraction and stabilization.

Beyond the CdS system investigated here, the PDI-PEI-carbon concept offers a modular interfacial strategy that could be extended to other semiconductor systems compatible with PEI-mediated coordination and growth. More generally, the assembly approach presented in this work provides a practical framework for constructing carbon-semiconductor hybrids under comparable conditions, facilitating more rigorous structure–function correlations.

## Figures and Tables

**Table 1:** Mass fractions of nitrogen, hydrogen, oxygen and sulfur in rGO-PEI, GNP-PDI-PEI and G-Sheet-PDI-PEI as determined by combustion elemental analysis (the oxygen content was calculated by difference).

Material	N (wt%)	H (wt%)	S (wt%)	C (wt%)	O (wt%) ^1^
RGO-PEI	10.7	3.6	1.0	62.3	22.4
GNP-PDI-PEI	6.3	3.0	0.0	75.3	15.4
G-Sheet-PDI-PEI	0.4	0.2	0.0	96.1	3.3

^1^ Oxygen content was calculated by difference from the remaining elemental composition.

**Table 2:** Atomic fractions of nitrogen, carbon, sulfur and oxygen in rGO-PEI, GNP-PDI-PEI and G-Sheet-PDI-PEI as determined by XPS analysis.

Material	N (at%)	C (at%)	S (at%)	O (at%)
RGO-PEI	10.9	68.9	1.0	19.2
GNP-PDI-PEI	6.5	85.3	0.0	8.2
G-Sheet-PDI-PEI	3.9	92.4	0.0	3.7

## Data Availability

All information examined in this research is incorporated within this article.

## Supplementary Materials

The following supporting information can be downloaded at: https://www.mdpi.com/article/10.3390/molecules31111920/s1, Figure S1: XPS spectra of (A) G-Sheet, (B) GNP and (C) GO; Figure S2: XPS spectra of (A) PDI-PEI, (B) G-Sheet-PDI-PEI, (C) GNP-PDI-PEI and (D) rGO-PEI; Figure S3: Experimental XRD pattern of rGO-PEI@CdS-500 compared with the simulated hexagonal CdS reference pattern. The indexed reflections correspond to wurtzite CdS, with the reflections associated with the (1 0 X) family highlighted in yellow; Figure S4: Tauc plots of [F(R)·hν]^2^ vs. photon energy for the hybrid materials, showing the determination of the optical band gap using the intersection method: (A) G-Sheet-PDI-PEI@CdS, (B) GNP-PDI-PEI@CdS, (C) rGO-PEI@CdS and (D) rGO-PEI@CdS-500. The extracted band gap values are 2.44, 2.48, 2.45, and 2.32 eV, respectively; Figure S5: Complementary optical microscopy and Raman analysis of rGO-PEI@CdS-500 before and after photocatalytic hydrogen evolution. Optical microscopy images of the pristine thermally treated sample were collected using (A) 5× and (B) 20× objectives. After 24 h of hydrogen evolution, the catalyst was recovered by filtration and dried at 120 °C for 2 h prior to analysis; optical microscopy images of the material were collected using (D) 5× and (E) 20× objectives. Representative Raman spectra recorded under 532 nm excitation are shown (C) before irradiation and (F) after photocatalysis; Figure S6: Thermogravimetric analysis of PEI-1.8K; Table S1: Atomic N/C ratios determined by XPS and elemental analysis for rGO-PEI, GNP-PDI-PEI and G-Sheet-PDI-PEI; Table S2: Hydrogen evolution data and solar-to-chemical conversion efficiency under simulated AM 1.5G irradiation.

## Abbreviations

The following abbreviations are used in this manuscript

GO	Graphene oxide
PEI	Hyperbranched polyethyleneimine
GNP	Graphene nanoplatelet
G-Sheet	Low-defect graphene sheet
PDI	Perylenediimide
