# Supplementary material for: Boosting Hydrogen Photogeneration via Controlled CdS Nucleation on PEI-Modified Graphene Surfaces
Source: Molecules. 2026 Jun 2;31(11):1920. doi: 10.3390/molecules31111920 (PMC13258516; doi:10.3390/molecules31111920)
Supplement: Supplementary file 1 [file molecules-31-01920-s001.zip › molecules-4322880-supplementary.pdf]

Supplementary material

Boosting Hydrogen Photogeneration via Controlled CdS Nucleation on PEI-Modified Graphene Surfaces

Jose J. Chica-Armenteros[1], Joan Vernet-García, Rubén Cruz-Sánchez, Celeste García-Gallarín, Antonio Peñas-Sanjuán and Manuel Melguizo**

[1]Departamento de Química Inorgánica y Orgánica. Facultad de Ciencias Experimentales, Universidad de Jaén, 23071 Jaén, Spain

*Corresponding authors

List of Figure, Table and Equation captions

XPS and combustion elemental analysis

Table S1. Atomic N/C ratios determined by XPS and elemental analysis for rGO-PEI, GNP-PDI-PEI and G-Sheet-PDI-PEI

Figure S1. XPS spectra of A) G-Sheet, B) GNP and C) GO

Figure S2. XPS spectra of A) PDI-PEI, B) G-Sheet-PDI-PEI, C) GNP-PDI-PEI and D) rGO-PEI

XRD pattern of rGO-PEI@CdS-500 compared to theoretical wurtzite diffraction pattern.

Figure S3. Experimental XRD pattern of rGO-PEI@CdS-500 compared with the simulated hexagonal CdS reference pattern. The indexed reflections correspond to wurtzite CdS, with the reflections associated with the (1 0 X) family highlighted in yellow

Determination of the optical band gap

Figure S4. Tauc plots of $[F(R)\cdot h\nu]^2$ vs photon energy for the hybrid materials, showing the determination of the optical band gap using the intersection method: A) G-Sheet-PDI-PEI@CdS, B) GNP-PDI-PEI@CdS, C) rGO-PEI@CdS and D) rGO-PEI@CdS-500. The extracted band gap values are 2.44, 2.48, 2.45, and 2.32 eV, respectively

Estimation of the solar-to-chemical conversion efficiency (SCC)

Table S2. Hydrogen evolution data and solar-to-chemical conversion efficiency under simulated AM 1.5G irradiation

Optical microscopy and Raman analysis of rGO-PEI@CdS-500

Figure S5. Complementary optical microscopy and Raman analysis of rGO-PEI@CdS-500 before and after photocatalytic hydrogen evolution. Optical microscopy images of the pristine thermally treated sample were collected using A) 5x and B) 20x objectives. After 24 h of hydrogen evolution, the catalyst was recovered by filtration and dried at 120 °C for 2 h prior to analysis; optical microscopy images of the material were collected using D) 5x and E) 20x objectives. Representative Raman spectra recorded under 532 nm excitation are shown C) before irradiation and F) after photocatalysis

Thermal analysis of PEI-1.8K

Figure S6. Thermogravimetric analysis of PEI-1.8K

XPS and combustion elemental analysis

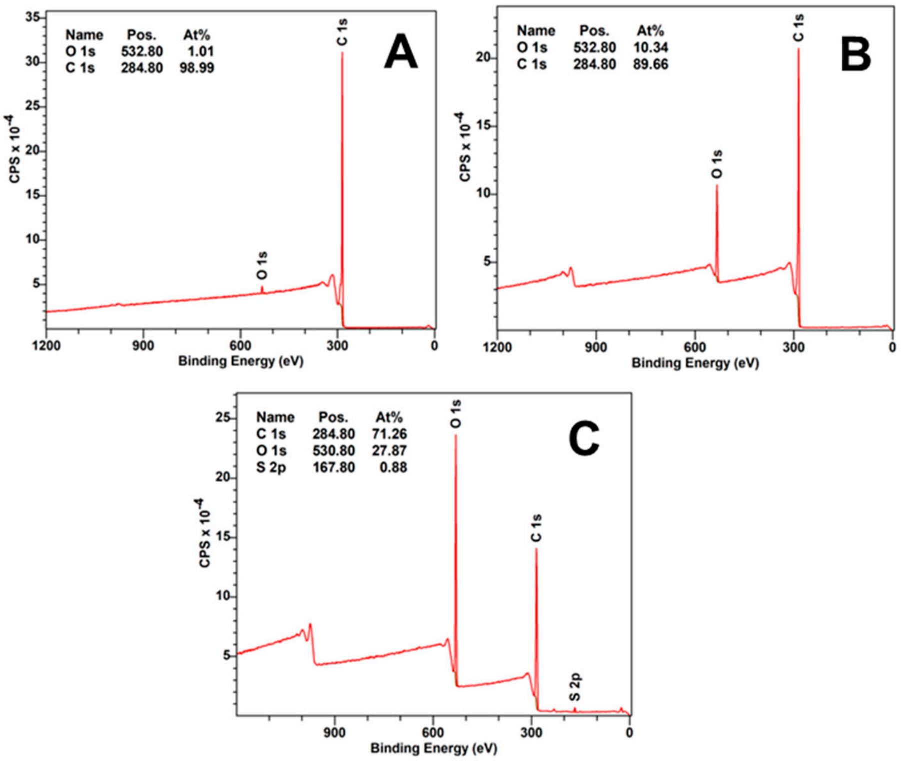

Figure S1. XPS spectra of A) G-Sheet, B) GNP and C) GO

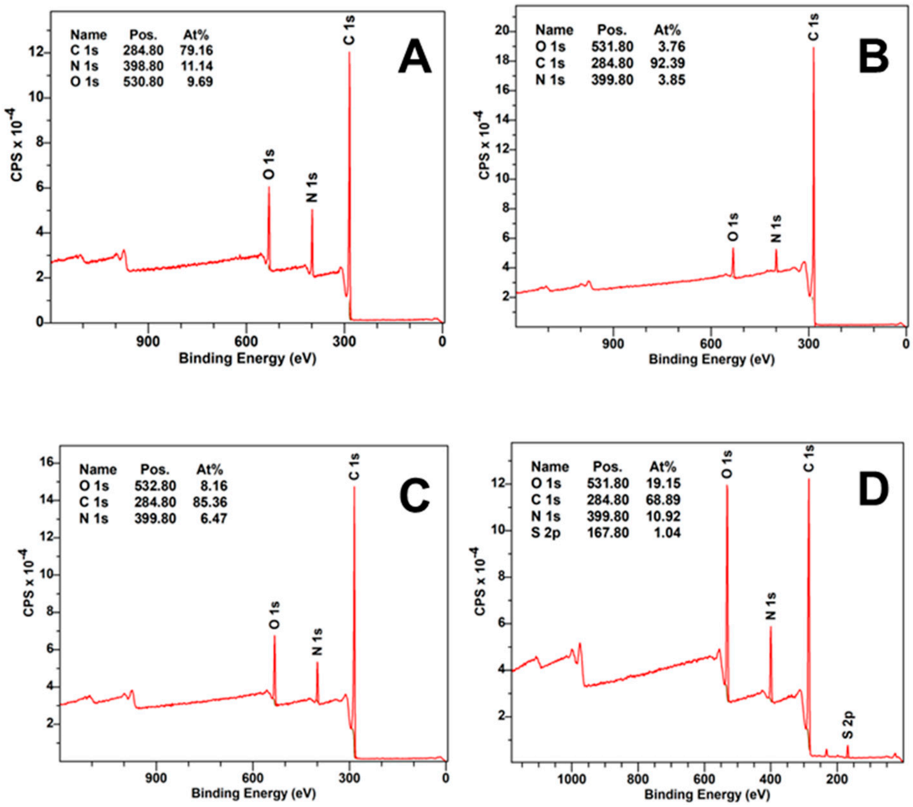

Figure S2. XPS spectra of A) PDI-PEI, B) G-Sheet-PDI-PEI, C) GNP-PDI-PEI and D) rGO-PEI

Table S1. Atomic N/C ratios determined by XPS and combustion elemental analysis for rGO-PEI, GNP-PDI-PEI and G-Sheet-PDI-PEI

Material	Elemental analysis			XPS		
	N (wt%)	C (wt%)	N/C[1]	N (at%)	C (at%)	N/C
rGO-PEI	10.7	62.3	0.147	10.9	68.9	0.159
GNP-PDI-PEI	6.3	75.3	0.072	6.5	85.3	0.076
G-Sheet-PDI-PEI	0.4	96.1	0.004	3.9	92.4.	0.042

[1]The N/C ratio was calculated from elemental analysis by converting mass percentages into molar amounts

XRD pattern of rGO-PEI@CdS-500 compared to theoretical wurtzite diffraction pattern.

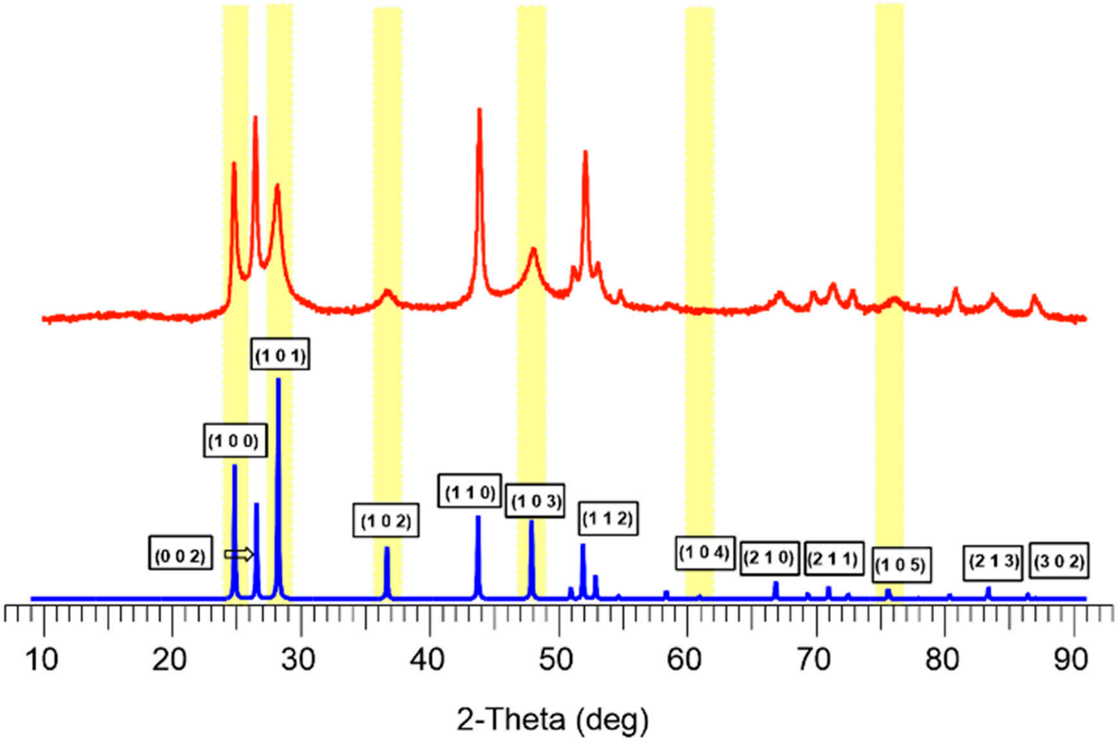

Figure S3. Experimental XRD pattern of rGO-PEI@CdS-500 compared with the simulated hexagonal CdS reference pattern. The indexed reflections correspond to wurtzite CdS, with the reflections associated with the (1 0 X) family highlighted in yellow

Determination of the optical band gap

The optical band gap of the different hybrid materials was determined from diffuse reflectance UV-Vis spectra using the Kubelka-Munk approximation[1,2]. The reflectance data (R) were transformed into the Kubelka-Munk function, F(R), according to:

$$F(R) = \frac{(1-R)^2}{2R} \quad (1)$$

where R corresponds to the measured diffuse reflectance of the sample.

Assuming a direct allowed electronic transition, the band gap energy (Eg) was estimated by constructing Tauc plots of $[F(R)\cdot h\nu]^2$ as a function of the photon energy ($h\nu$). Eg was determined using the intersection method, following the approach described in the literature[3]. In this method, two linear regions are considered: (i) the linear portion associated with the fundamental absorption edge and (ii) the baseline in the sub-bandgap region. The band gap value is obtained from the intersection of these two linear fits. The corresponding Tauc plots together with the linear fittings used for band gap determination are presented in Figure S4.

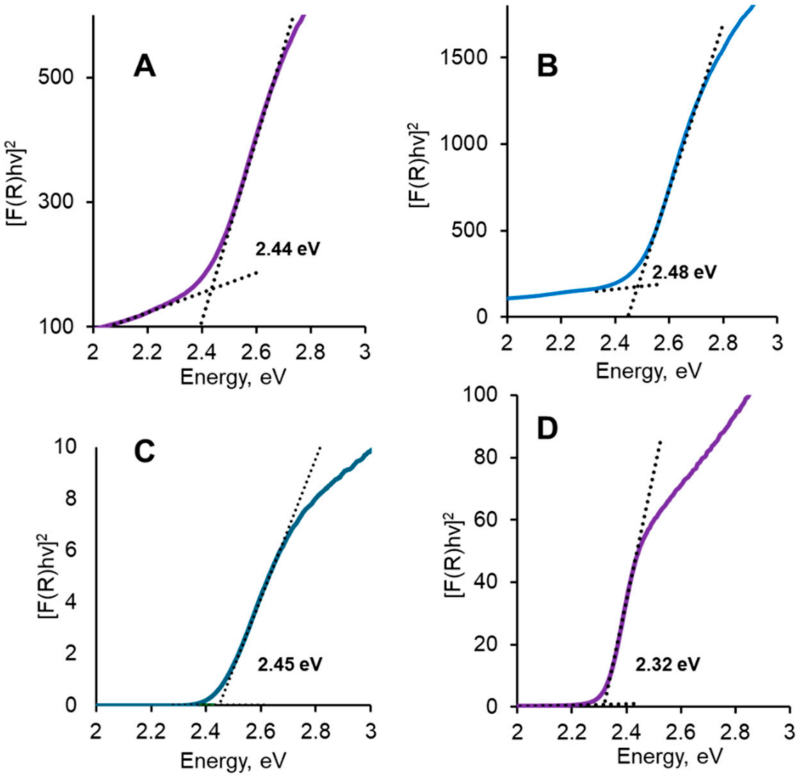

Figure S4. Tauc plots of $[F(R)\cdot h\nu]^2$ vs photon energy for the hybrid materials, showing the determination of the optical band gap using the intersection method: A) G-Sheet-PDI-PEI@CdS, B)

GNP-PDI-PEI@CdS, C) rGO-PEI@CdS and D) rGO-PEI@CdS-500. The extracted band gap values are 2.44, 2.48, 2.45, and 2.32 eV, respectively

Estimation of the solar-to-chemical conversion efficiency (SCC)

The solar-to-chemical conversion efficiency for sacrificial hydrogen evolution, SCC, was estimated under simulated AM 1.5G irradiation as a comparative metric among samples measured under identical sacrificial photocatalytic conditions. SCC was calculated as the ratio between the reference chemical energy associated with H_2 formation under sacrificial conditions and the incident solar energy, according to:

$$SCC(\%) = \frac{r_{H_2} \cdot \Delta G_{H_{ER}}}{S_{ir} \cdot I_{AM}} \cdot 100 (2)$$

Where $\Delta G_{H_{ER}}$ is the effective Gibbs free energy associated with H_2 evolution coupled to the oxidation of the sacrificial redox system, r_{H_2} is the H_2 evolution rate expressed in mol·s^{-1} (Table S2), S_{ir} is the irradiated area, 3.14 cm^2, and I_{AM} is the incident irradiance under AM 1.5G conditions, 0.1 W·cm^{-2}.

In this work, $\Delta G_{H_{ER}}$ was defined considering that H_2 evolution is coupled to oxidation of the sacrificial redox system, Na_2S/Na_2SO_3, rather than to O_2 evolution [4]. Therefore, the $\Delta G_{H_{ER}}$ was taken from the value proposed in Ref [4] corresponding to 90695 J·mol^{-1}.

Table S2. Hydrogen evolution data and solar-to-chemical conversion efficiency under simulated AM 1.5G irradiation

**Material**	$r_{H_2}/10^{-10}$ mol·s^{-1}	SCC (%)
G-Sheet-PDI-PEI	2.50	0.007
GNP-PDI-PEI	4.17	0.012
rGO-PEI	6.11	0.018
rGO-PEI-500	34.7[a] ;11.5[b]	0.100[a] and 0.033[b]

[a] Calculated from the initial hydrogen evolution rate in the 0-5 h interval.

[b] Calculated from the hydrogen evolution rate in the 5-24 h interval.

Optical microscopy and Raman analysis of rGO-PEI@CdS-500

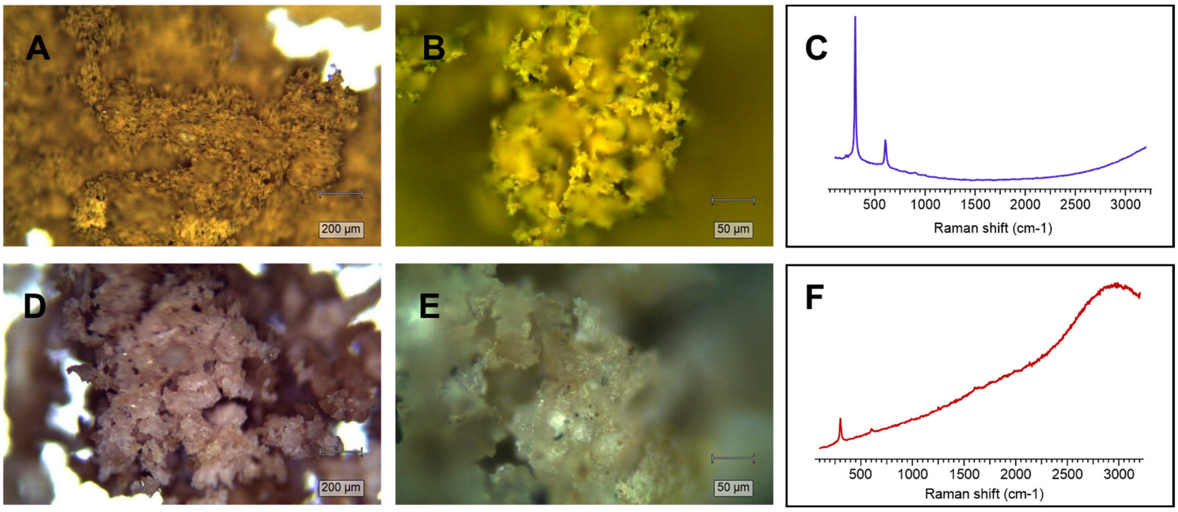

Figure S5. Complementary optical microscopy and Raman analysis of rGO-PEI@CdS-500 before and after photocatalytic hydrogen evolution. Optical microscopy images of the pristine thermally treated sample were collected using A) 5x and B) 20x objectives. After 24 h of hydrogen evolution, the catalyst was recovered by filtration and dried at 120 °C for 2 h prior to analysis; optical microscopy images of the material were collected using D) 5x and E) 20x objectives. Representative Raman spectra recorder under 532 nm excitation are shown C) before irradiation and F) after photocatalysis

Thermal analysis of PEI-1.8K

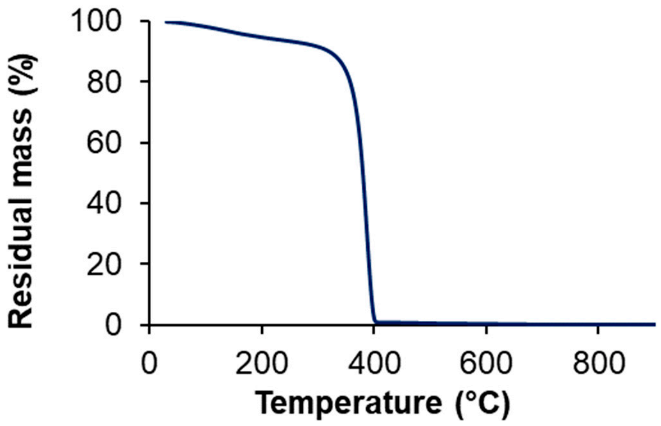

Figure S6. Thermogravimetric analysis of PEI-1.8K

References

1. Murphy, A.B. Band-Gap Determination from Diffuse Reflectance Measurements of Semiconductor Films, and Application to Photoelectrochemical Water-Splitting. *Sol. Energy Mater. Sol. Cells* **2007**, *91*, 1326–1337, doi:https://doi.org/10.1016/j.solmat.2007.05.005.

2. López, R.; Gómez, R. Band-Gap Energy Estimation from Diffuse Reflectance Measurements on Sol–Gel and Commercial TiO2: A Comparative Study. *J. Sol-Gel Sci. Technol.* **2012**, *61*, 1–7, doi:10.1007/s10971-011-2582-9.

3. Makuła, P.; Pacia, M.; Macyk, W. How To Correctly Determine the Band Gap Energy of Modified Semiconductor Photocatalysts Based on UV–Vis Spectra. *J. Phys. Chem. Lett.* **2018**, *9*, 6814–6817, doi:10.1021/acs.jpclett.8b02892.

4. Holmes, A.; Wang, E. Toward Standardized and Comparable Reporting for Sacrificial Photocatalytic Hydrogen Evolution. *ACS Energy Lett.* **2026**, doi:10.1021/acsenergylett.6c00531.
